# Supplementary figures and images for: Genetic Variation of Promoter Sequence Modulates XBP1 Expression and Genetic Risk for Vitiligo
Source: PLoS Genet. 2009 Jun 19;5(6):e1000523. doi: 10.1371/journal.pgen.1000523 (PMC2689933; doi:10.1371/journal.pgen.1000523)

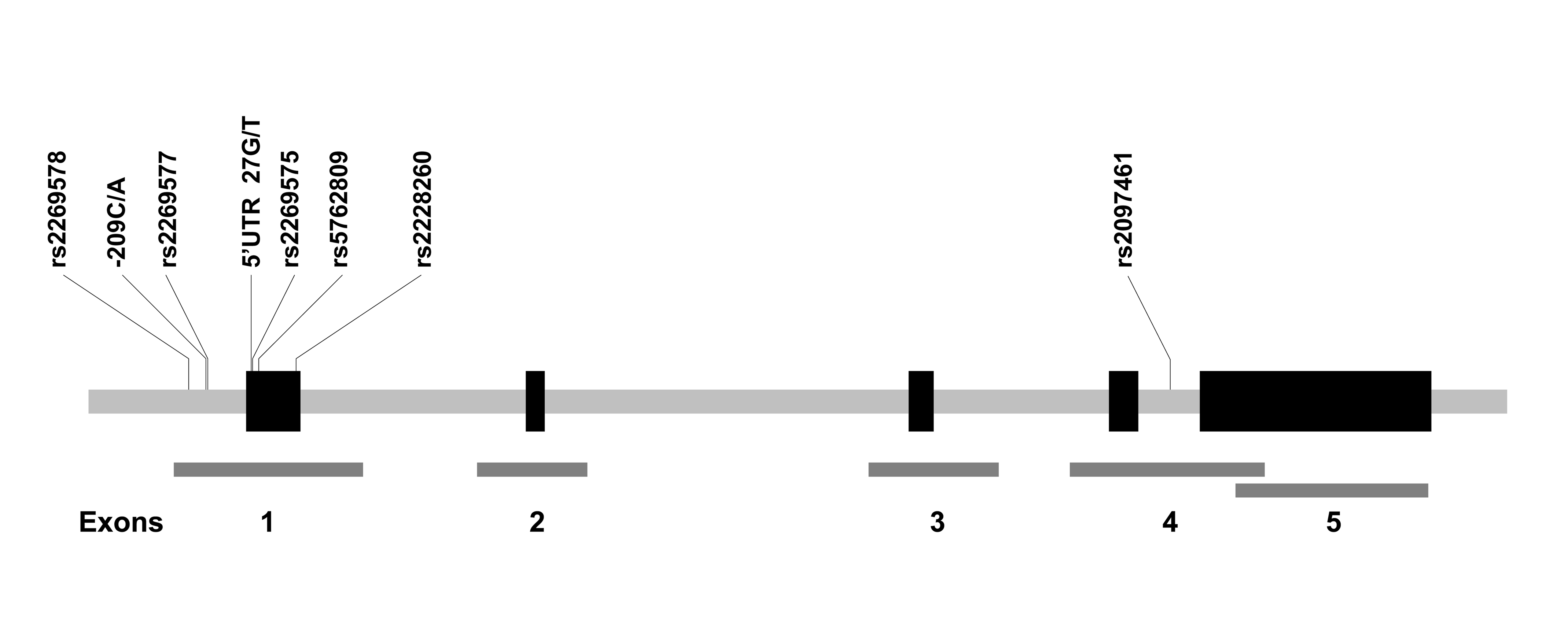

Supplement: Figure S1 — Genomic structure of the XBP1 gene with the location of 8 SNPs subjected to genetic association analysis. The gray bars (underneath the genomic structure) indicate the genomic regions that were amplified for sequencing analysis. (1.34 MB TIF) [file pgen.1000523.s001.tif]
