# Supplementary material for: Genetic Variation of Promoter Sequence Modulates XBP1 Expression and Genetic Risk for Vitiligo
Source: PLoS Genet. 2009 Jun 19;5(6):e1000523. doi: 10.1371/journal.pgen.1000523 (PMC2689933; doi:10.1371/journal.pgen.1000523)
Supplement: Table S1 — A list of all the variants identified by the initial sequencing analysis of XBP1. (0.07 MB DOC) [file pgen.1000523.s002.doc]

**Table S1**.

A list of all the variants identified by the initial sequencing analysis of XBP1.

| SNP | Position | | Location | Allele | Freq | # Carriers |
| --- | --- | --- | --- | --- | --- | --- |
| rs2269578 | | 8587412 | promoter, 283bp upstream of exon 1 | G/C | 0.20 |  |
| XBP1snp1 | | 8587338 | promoter, 209bp upstream of exon 1 | A/C | 0.03 |  |
| rs2269577 | | 8587326 | promoter, 197bp upstream of exon 1 | C/G | 0.38 |  |
| rs2269576 | | 8587316 | promoter, 187bp upstream of exon 1 | G/C |  | three subjects |
| XBP1snp2 | | 8587315 | promoter, 186bp upstream of exon 1 | A/G |  | one subject |
| XBP1snp3 | | 8587304 | promoter,175bp upstream of exon 1 | T/C |  | one subject |
| XBP1snp4 | | 8587266 | promoter, 137bp upstream of exon 1 | G/C |  | one subject |
| XBP1snp5 | | 8587234 | promoter, 105bp upstream of exon 1 | A/G |  | one subject |
| XBP1snp6 | | 8587155 | promoter, 26bp upstream of exon 1 | C/T |  | two subjects |
| XBP1snp7 | | 8587143 | promoter, 14bp upstream of exon 1 | delG |  | one subject |
| XBP1snp8 | | 8587113 | exon 1, 17bp 5'UTR | C/G | 0.01 |  |
| XBP1snp9 | | 8587106 | exon 1, 24bp 5'UTR | A/G |  | two subjects |
| XBP1snp10 | | 8587103 | exon 1, 27bp 5'UTR | T/G | 0.06 |  |
| XBP1snp11 | | 8587100 | exon 1, 30bp 5'UTR | T/C |  | one subject |
| rs2269575 | | 8587095 | exon 1, 35bp 5'UTR | T/C | 0.27 |  |
| XBP1snp12 | | 8587067 | exon 1, | insGGC |  | one family |
| rs5762809 | | 8587063 | exon 1, non-synonymous (A7S) | A/G | 0.19 |  |
| XBP1snp13 | | 8587001 | exon 1, synonymous | T/C |  | one subject |
| rs2228260 | | 8586875 | exon 1, synonymous | T/C | 0.08 |  |
| XBP1snp14 | | 8585756 | intron1 | G/A |  | one subject |
| XBP1snp15 | | 8585738 | intron1 | A/C |  | one subject |
| XBP1snp16 | | 8585608 | intron2 | G/A |  | one subject |
| XBP1snp17 | | 8583778 | intron2 | G/A |  | one subject |
| XBP1snp18 | | 8583737 | exon3,non-synonymous (Q117R) | G/A |  | one subject |
| XBP1snp19 | | 8583576 | intron3 | G/C |  | one subject |
| XBP1snp20 | | 8583504 | intron3 | C/T |  | two subjects |
| XBP1snp21 | | 8582868 | intron3 | A/G |  | one subject |
| rs35873774 | | 8582501 | intron4 | C/T |  | two subjects |
| rs34842534 | | 8582467 | intron4 | T/C |  | one subject |
| rs2097461 | | 8582448 | intron4 | A/G | 0.40 |  |
| XBP1snp22 | | 8582405 | intron4 | T/G |  | one subject |
| XBP1snp23 | | 8582384 | intron4 | T/C |  | one subject |
| XBP1snp24 | | 8581721 | exon 5, 11bp 3' UTR | G/A |  | one subject |
| XBP1snp25 | | 8581533 | exon 5, 199bp 3' UTR | C/T | 0.01 |  |
