# Supplementary material for: Genetic Variation of Promoter Sequence Modulates XBP1 Expression and Genetic Risk for Vitiligo
Source: PLoS Genet. 2009 Jun 19;5(6):e1000523. doi: 10.1371/journal.pgen.1000523 (PMC2689933; doi:10.1371/journal.pgen.1000523)
Supplement: Table S2 — Primers used for the sequencing analysis of the exons, exon-intron boundaries, and some promoter sequences of XBP1. (0.03 MB DOC) [file pgen.1000523.s003.doc]

**Table S2**.

Primers used for the sequencing analysis of the exons, exon-intron boundaries and some promoter sequences of the XBP1.

| Target | Forward primer | Reverse primer | Fragment size |
| --- | --- | --- | --- |
| Exon1 | ggcgcacgagataaaatgttg | ccgagttaagaggctgaacca | 968bp |
| Exon2 | gggatgagtctgaggtggac | ggtctcaaactgctggcttc | 563 bp |
| Exon3 | ttcttgaccccatgaagtgg | gcgttcaaaatctggtttgag | 668 bp |
| Exon4 | gattcaagcagaactggcaaa | ggttcttccttcactgagacaa | 997 bp |
| Exon5 | gtcatcagccaagctggaa | tggagaaagcacctttcaga | 981 bp |
