# Supplementary material for: Genetic Variation of Promoter Sequence Modulates XBP1 Expression and Genetic Risk for Vitiligo
Source: PLoS Genet. 2009 Jun 19;5(6):e1000523. doi: 10.1371/journal.pgen.1000523 (PMC2689933; doi:10.1371/journal.pgen.1000523)
Supplement: Table S3 — Frequencies of HLA-DR alleles in vitiligo patients compared with control group. (0.03 MB DOC) [file pgen.1000523.s004.doc]

**Table S3.**

Frequencies of HLA-DR alleles in vitiligo patients compared with control group.

| HLA-DR alleles | Allele frequency | | P-value | OR(95% CI) |
| --- | --- | --- | --- | --- |
|  | Patient | Control |  |  |
| DRB1*04 | 0.13 | 0.14 | 0.35 | 0.92(0.77-1.10) |
| DRB1*07 | 0.21 | 0.13 | 4.53E-21 | 1.98(1.72-2.29) |
| DRB1*12 | 0.20 | 0.19 | 0.29 | 1.09(0.93-1.29) |
| DRB4*01 | 0.42 | 0.41 | 0.16 | 1.17(0.94-1.45) |
